# {2-[(4-Nitro­benzyl­idene)amino]-4,5,6,7-tetrahydro-1-benzo­thio­phen-3-yl}(phen­yl)methanone

**DOI:** 10.1107/S1600536814012185

**Published:** 2014-05-31

**Authors:** Manpreet Kaur, Jerry P. Jasinski, Channappa N. Kavitha, H. S. Yathirajan, K. Byrappa

**Affiliations:** a Department of Studies in Chemistry, University of Mysore, Manasagangotri, Mysore 570 006, India; b Department of Chemistry, Keene State College, 229 Main Street, Keene, NH 03435-2001, USA; c Materials Science Center, University of Mysore, Vijyana Bhavan Building, Manasagangothri, Mysore 570 006, India

## Abstract

In the title compound, C_22_H_18_N_2_O_3_S, disorder is found in the benzoyl group (*A* and *B*), as well as for four C atoms of the cyclo­hexene ring. Two orientations were modeled in a 0.583 (5):0.417 (5) ratio. The cyclo­hexene ring is in a distorted chair conformation. The dihedral angles between the mean plane of the thio­phene ring and the 4-nitro­benzene and phenyl rings are 30.9 (8) and 64.8 (3) (*A*) and 62.4 (7)° (*B*). The mean planes of the 4-nitro­benzene and the phenyl rings are almost perpendicular to each other, with dihedral angles of 85.4 (1) (*A*) and 83.9 (8)° (*B*). An extensive array of weak C—H⋯O inter­actions consolidate mol­ecules into a three-dimensional architecture, forming chains along [001] and [010] and layers parallel to (011).

## Related literature   

For applications of 2-amino­thio­phene derivatives in pesticides, dyes and pharmaceuticals, see: Puterová *et al.* (2010 ▶). For the biological and industrial importance of Schiff bases, see: Desai *et al.* (2001 ▶); Karia & Parsania (1999 ▶); Samadhiya & Halve (2001 ▶); Singh & Dash (1988 ▶). For Schiff bases utilized as starting materials in the synthesis of compounds of industrial and biological inter­est, see: Aydogan *et al.* (2001 ▶); Taggi *et al.* (2002 ▶). For related structures, see: Kaur *et al.* (2014*a* ▶,*b* ▶); Kubicki *et al.* (2012 ▶). For puckering parameters, see: Cremer & Pople (1975 ▶).
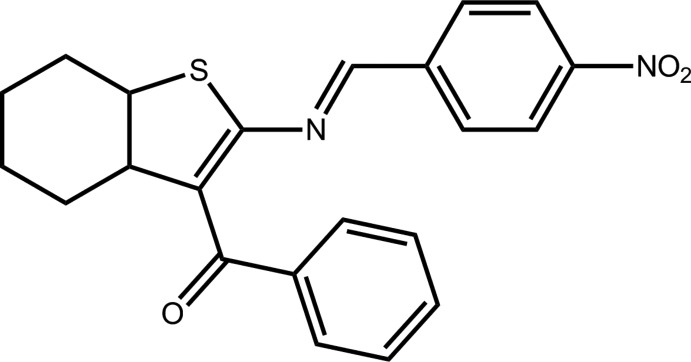


## Experimental   

### 

#### Crystal data  

C_22_H_18_N_2_O_3_S
*M* *_r_* = 390.44
Monoclinic, 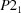

*a* = 4.61595 (13) Å
*b* = 17.6844 (4) Å
*c* = 11.7068 (3) Å
β = 91.285 (3)°
*V* = 955.39 (4) Å^3^
*Z* = 2
Cu *K*α radiation
μ = 1.72 mm^−1^
*T* = 173 K
0.24 × 0.18 × 0.06 mm

#### Data collection  

Agilent Eos Gemini diffractometer
Absorption correction: multi-scan (*CrysAlis PRO* and *CrysAlis RED*; Agilent, 2012 ▶) *T*_min_ = 0.634, *T*_max_ = 1.000
6206 measured reflections
2781 independent reflections
2610 reflections with *I* > 2σ(*I*)
*R*_int_ = 0.027

#### Refinement  

*R*[*F* ^2^ > 2σ(*F* ^2^)] = 0.035
*wR*(*F* ^2^) = 0.090
*S* = 1.02
2781 reflections
263 parameters
138 restraints
H-atom parameters constrained
Δρ_max_ = 0.21 e Å^−3^
Δρ_min_ = −0.19 e Å^−3^
Absolute structure: Flack *x* determined using 764 quotients [(*I* ^+^)−(*I* ^−^)]/[(*I* ^+^)+(*I* ^−^)] (Parsons & Flack, 2004 ▶)
Absolute structure parameter: 0.028 (16)

### 

Data collection: *CrysAlis PRO* (Agilent, 2012 ▶); cell refinement: *CrysAlis PRO*; data reduction: *CrysAlis RED* (Agilent, 2012 ▶); program(s) used to solve structure: *SUPERFLIP* (Palatinus & Chapuis, 2007 ▶); program(s) used to refine structure: *SHELXL2012* (Sheldrick, 2008 ▶); molecular graphics: *OLEX2* (Dolomanov *et al.*, 2009 ▶); software used to prepare material for publication: *OLEX2*.

## Supplementary Material

Crystal structure: contains datablock(s) I. DOI: 10.1107/S1600536814012185/tk5318sup1.cif

Structure factors: contains datablock(s) I. DOI: 10.1107/S1600536814012185/tk5318Isup2.hkl

Supporting information file. DOI: 10.1107/S1600536814012185/tk5318Isup3.cml

CCDC reference: 1005353

Additional supporting information:  crystallographic information; 3D view; checkCIF report

## Figures and Tables

**Table 1:** Hydrogen-bond geometry (Å, °)

*D*—H⋯*A*	*D*—H	H⋯*A*	*D*⋯*A*	*D*—H⋯*A*
C4*A*—H4*AA*⋯O1*B* ^i^	0.99	2.38	3.15 (4)	135
C4*B*—H4*BA*⋯O1*A* ^i^	0.99	2.50	3.45 (4)	162
C4*B*—H4*BA*⋯O1*B* ^i^	0.99	2.26	3.18 (4)	154
C7*B*—H7*BB*⋯O2^ii^	0.99	2.46	3.44 (3)	169
C13*B*—H13*B*⋯O3^iii^	0.95	2.55	3.371 (13)	145
C21—H21⋯O1*A* ^iv^	0.95	2.40	3.127 (18)	133
C21—H21⋯O1*B* ^iv^	0.95	2.44	3.13 (3)	129

Symmetry codes: (i) 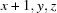; (ii) 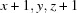; (iii) 
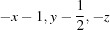
; (iv) 
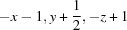
.

**Fig. 1.**
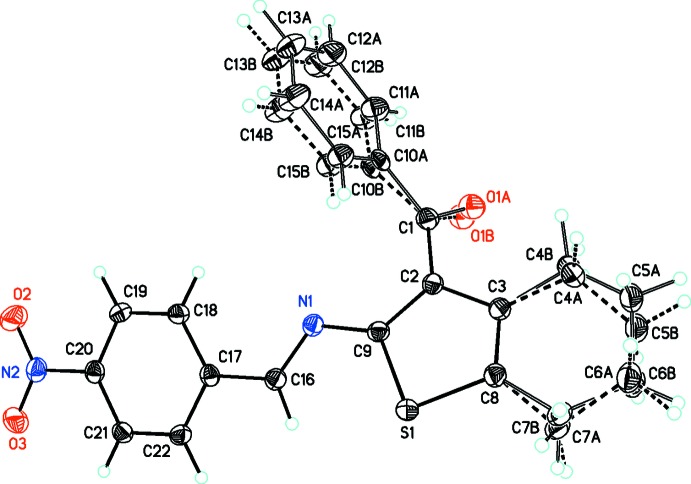
ORTEP drawing of (I) showing the labeling scheme and 30% probability displacement ellipsoids (major and minor components of the disordered atoms in the cyclohexane, benzoyl groups are displayed with dashed lines).

**Fig. 2.**
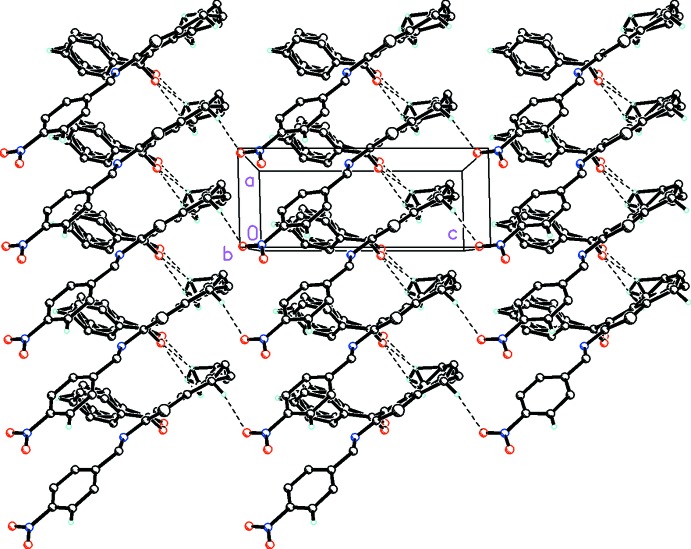
Molecular packing for (I) viewed along the b axis. Dashed lines indicate weak C—H···O intermolecular interactions. H atoms not involved in the weak intermolecular interactions have been removed for clarity.

## supplementary crystallographic information

### 2. Structural commentary

2-Amino­thio­phene derivatives have been used in a number of applications, such as in pesticides, dyes and pharmaceuticals. A review on the synthesis and properties of these compounds has been reported (Puterová *et al.*, 2010). Schiff base compounds are an important class of compounds, both synthetically and biologically. These compounds show biological activities including anti-bacterial, anti-fungal, anti-cancer and herbicidal (Desai *et al.*, 2001; Karia & Parsania, 1999; Samadhiya & Halve, 2001; Singh & Dash, 1988). Furthermore, Schiff bases are utilized as starting materials in the synthesis of compounds of industrial (Aydogan *et al.*, 2001) and biological inter­est such as β-la­ctams (Taggi *et al.*, 2002). Some of the recently reported Schiff base structures of 2-amino­thio­phenes by our group include {2-[(2-hy­droxy­benzyl­idene)amino]-4,5,6,7-tetra­hydro-1-benzo­thio­phene-3-yl} (phenyl)­methanone (Kaur *et al.*, 2014*a*) and [2-benzyl­idene­amino)-4,5,6,7-tetra­hydro benzo[b]thio­phene-3-yl](phenyl) methanone (Kaur *et al.*, 2014*b*). Also, the crystal and molecular structures of two 2-amino­thio­phenes have been previously reported by our group (Kubicki *et al.*, 2012). In continuation of our work on 2-amino­thio­phenes and Schiff bases, we report here the crystal structure of the title compound, (I).

In (I), the cyclo­hexene ring is in a distorted chair conformation with four carbon atoms disordered over two sets of sites with an occupancy ratio of 0.583 (5): 0.417 (5) (Fig. 1). Puckering parameters C3/C4A—C7A/C8: Q and φ = 0.511 (4) Å and 157.387 (6)°, and C3/C4B—C7B/C8: Q and φ = 0.483 (8) Å and 212.306 (8)° (Cremer & Pople, 1975). The disorder extends to the benzoyl residue (A & B). The dihedral angles between the mean plane of the thio­phene ring and the 4-nitro­phenyl and the phenyl rings is 30.9 (8) and 64.8 (3) (A) and 62.4 (7)° (B), respectively. The mean planes of 4-nitro­phenyl and the phenyl rings are almost perpendicular to each other with a dihedral angle of 85.4 (1) (A) and 83.9 (8)° (B). An extensive array of weak C—H···O inter­molecular inter­actions leads to a 3-D architecure, forming chains along [001] and [010] and layers parallel to (011) (Fig. 2).

### 5. Synthesis and crystallization

To a solution of (2-amino-4,5,6,7-tetra­hydro-benzo[b]thio­phen-3-yl)- phenyl-methanone (200 mg, 0.79 mmol) in 10 ml of methanol an equimolar amount of 4-nitro­benzaldehyde (120 mg, 0.79 mmol) was added with constant stirring. The mixture was refluxed for 3 h. A yellow precipitate was obtained. Completion of the reaction was confirmed by TLC. The precipitate was filtered and dried at room temperature overnight. The solid was recrystallized using ethyl­acetate and the crystals were used as such for x-ray diffraction studies.

### 6. Refinement

The H atoms were placed in their calculated positions and then refined using the riding model with Atom—H lengths of 0.93 Å (CH) or 0.97 Å (CH_2_), and with U_iso_ set to 1.2*U*_eq_ of the parent atom. Disorder was modeled for C4A—C7A and C4B—C7B of the cyclo­hexane ring, C10A—C15A/O1A and C10B—C15B/O1B of the benzoyl group over two positions with an occupancy ratio of 0.583 (5):0.417 (5).

### Crystal data

**Table:**

C_22_H_18_N_2_O_3_S	*F*(000) = 408
*M**_r_* = 390.44	*D*_x_ = 1.357 Mg m^−^^3^
Monoclinic, *P*2_1_	Cu *K*α radiation, λ = 1.54184 Å
*a* = 4.61595 (13) Å	Cell parameters from 3146 reflections
*b* = 17.6844 (4) Å	θ = 4.5–71.4°
*c* = 11.7068 (3) Å	µ = 1.72 mm^−^^1^
β = 91.285 (3)°	*T* = 173 K
*V* = 955.39 (4) Å^3^	Irregular, yellow
*Z* = 2	0.24 × 0.18 × 0.06 mm

### Data collection

**Table:**

Agilent Eos Gemini diffractometer	2781 independent reflections
Radiation source: Enhance (Cu) X-ray Source	2610 reflections with *I* > 2σ(*I*)
Detector resolution: 16.0416 pixels mm^-1^	*R*_int_ = 0.027
ω scans	θ_max_ = 71.1°, θ_min_ = 3.8°
Absorption correction: multi-scan (*CrysAlis PRO* and *CrysAlis RED*; Agilent, 2012)	*h* = −5→5
*T*_min_ = 0.634, *T*_max_ = 1.000	*k* = −18→21
6206 measured reflections	*l* = −11→14

### Refinement

**Table:**

Refinement on *F*^2^	Hydrogen site location: mixed
Least-squares matrix: full	H-atom parameters constrained
*R*[*F*^2^ > 2σ(*F*^2^)] = 0.035	*w* = 1/[σ^2^(*F*_o_^2^) + (0.0608*P*)^2^] where *P* = (*F*_o_^2^ + 2*F*_c_^2^)/3
*wR*(*F*^2^) = 0.090	(Δ/σ)_max_ < 0.001
*S* = 1.02	Δρ_max_ = 0.21 e Å^−^^3^
2781 reflections	Δρ_min_ = −0.19 e Å^−^^3^
263 parameters	Absolute structure: Flack *x* determined using 764 quotients [(*I*^+^)-(*I*^-^)]/[(*I*^+^)+(*I*^-^)] (Parsons & Flack, 2004)
138 restraints	Absolute structure parameter: 0.028 (16)
Primary atom site location: structure-invariant direct methods	

### Special details

**Table:**

Geometry. All esds (except the esd in the dihedral angle between two l.s. planes) are estimated using the full covariance matrix. The cell esds are taken into account individually in the estimation of esds in distances, angles and torsion angles; correlations between esds in cell parameters are only used when they are defined by crystal symmetry. An approximate (isotropic) treatment of cell esds is used for estimating esds involving l.s. planes.

### Fractional atomic coordinates and isotropic or equivalent isotropic displacement parameters (Å^2^)

**Table:**

	*x*	*y*	*z*	*U*_iso_*/*U*_eq_	Occ. (<1)
S1	0.24441 (14)	0.70387 (3)	0.62913 (5)	0.03214 (17)	
O1A	0.009 (2)	0.4397 (10)	0.5799 (16)	0.041 (2)	0.583 (5)
O1B	−0.071 (4)	0.4474 (15)	0.576 (2)	0.041 (2)	0.417 (5)
O2	−0.9787 (6)	0.75162 (18)	−0.0143 (2)	0.0605 (7)	
O3	−1.1148 (6)	0.84877 (16)	0.0802 (2)	0.0551 (7)	
N1	−0.0687 (5)	0.64901 (14)	0.44225 (18)	0.0293 (5)	
N2	−0.9655 (6)	0.79235 (16)	0.0691 (2)	0.0385 (6)	
C1	0.0964 (6)	0.49027 (16)	0.5199 (2)	0.0338 (6)	
C2	0.1876 (6)	0.56264 (16)	0.5745 (2)	0.0280 (5)	
C3	0.3469 (6)	0.56491 (16)	0.6813 (2)	0.0303 (6)	
C4A	0.439 (6)	0.4943 (15)	0.7449 (16)	0.0392 (15)	0.583 (5)
H4AA	0.5141	0.4559	0.6915	0.047*	0.583 (5)
H4AB	0.2683	0.4731	0.7832	0.047*	0.583 (5)
C5A	0.6700 (13)	0.5148 (4)	0.8363 (5)	0.0438 (11)	0.583 (5)
H5AA	0.7031	0.4720	0.8895	0.053*	0.583 (5)
H5AB	0.8537	0.5248	0.7969	0.053*	0.583 (5)
C6A	0.5854 (15)	0.5850 (4)	0.9033 (5)	0.0428 (12)	0.583 (5)
H6AA	0.3961	0.5767	0.9394	0.051*	0.583 (5)
H6AB	0.7322	0.5949	0.9644	0.051*	0.583 (5)
C7A	0.578 (5)	0.6541 (8)	0.8218 (18)	0.035 (2)	0.583 (5)
H7AA	0.4883	0.6968	0.8628	0.042*	0.583 (5)
H7AB	0.7767	0.6695	0.8023	0.042*	0.583 (5)
C4B	0.464 (8)	0.502 (2)	0.754 (2)	0.0392 (15)	0.417 (5)
H4BA	0.6486	0.4861	0.7197	0.047*	0.417 (5)
H4BB	0.3324	0.4574	0.7522	0.047*	0.417 (5)
C5B	0.5365 (19)	0.5254 (5)	0.8763 (7)	0.0438 (11)	0.417 (5)
H5BA	0.3503	0.5333	0.9149	0.053*	0.417 (5)
H5BB	0.6429	0.4844	0.9169	0.053*	0.417 (5)
C6B	0.717 (2)	0.5966 (5)	0.8813 (8)	0.0428 (12)	0.417 (5)
H6BA	0.7592	0.6094	0.9623	0.051*	0.417 (5)
H6BB	0.9050	0.5874	0.8443	0.051*	0.417 (5)
C7B	0.551 (7)	0.6653 (12)	0.830 (3)	0.035 (2)	0.417 (5)
H7BA	0.6977	0.7031	0.8078	0.042*	0.417 (5)
H7BB	0.4169	0.6895	0.8834	0.042*	0.417 (5)
C8	0.3991 (6)	0.63650 (17)	0.7187 (2)	0.0310 (6)	
C9	0.1092 (6)	0.63358 (15)	0.5361 (2)	0.0276 (6)	
C10A	0.1914 (17)	0.4696 (6)	0.4023 (7)	0.0261 (15)	0.583 (5)
C11A	0.0684 (16)	0.4049 (6)	0.3543 (9)	0.0430 (17)	0.583 (5)
H11A	−0.0653	0.3758	0.3964	0.052*	0.583 (5)
C12A	0.1411 (17)	0.3828 (5)	0.2446 (9)	0.049 (2)	0.583 (5)
H12A	0.0571	0.3386	0.2118	0.059*	0.583 (5)
C13A	0.3367 (16)	0.4254 (6)	0.1830 (7)	0.050 (2)	0.583 (5)
H13A	0.3864	0.4103	0.1081	0.060*	0.583 (5)
C14A	0.4597 (16)	0.4901 (5)	0.2311 (9)	0.051 (2)	0.583 (5)
H14A	0.5934	0.5192	0.1889	0.061*	0.583 (5)
C15A	0.3870 (18)	0.5122 (5)	0.3407 (9)	0.0347 (17)	0.583 (5)
H15A	0.4711	0.5564	0.3735	0.042*	0.583 (5)
C10B	0.127 (3)	0.4756 (10)	0.3952 (11)	0.0261 (15)	0.417 (5)
C11B	−0.001 (3)	0.4119 (9)	0.3461 (13)	0.0430 (17)	0.417 (5)
H11B	−0.1300	0.3819	0.3890	0.052*	0.417 (5)
C12B	0.060 (3)	0.3922 (7)	0.2342 (14)	0.049 (2)	0.417 (5)
H12B	−0.0276	0.3487	0.2007	0.059*	0.417 (5)
C13B	0.249 (3)	0.4360 (9)	0.1715 (11)	0.050 (2)	0.417 (5)
H13B	0.2906	0.4225	0.0950	0.060*	0.417 (5)
C14B	0.377 (3)	0.4997 (8)	0.2206 (13)	0.051 (2)	0.417 (5)
H14B	0.5065	0.5297	0.1777	0.061*	0.417 (5)
C15B	0.316 (3)	0.5194 (8)	0.3324 (14)	0.0347 (17)	0.417 (5)
H15B	0.4041	0.5629	0.3660	0.042*	0.417 (5)
C16	−0.1906 (5)	0.71396 (17)	0.4340 (2)	0.0295 (6)	
H16	−0.1542	0.7504	0.4921	0.035*	
C17	−0.3839 (5)	0.73363 (15)	0.3379 (2)	0.0277 (5)	
C18	−0.4040 (6)	0.68891 (15)	0.2402 (2)	0.0319 (6)	
H18	−0.2870	0.6449	0.2344	0.038*	
C19	−0.5926 (6)	0.70787 (19)	0.1514 (2)	0.0339 (6)	
H19	−0.6052	0.6778	0.0841	0.041*	
C20	−0.7634 (6)	0.77200 (16)	0.1630 (2)	0.0309 (6)	
C21	−0.7499 (6)	0.81762 (16)	0.2582 (2)	0.0310 (6)	
H21	−0.8692	0.8612	0.2637	0.037*	
C22	−0.5568 (6)	0.79807 (16)	0.3461 (2)	0.0316 (6)	
H22	−0.5423	0.8289	0.4124	0.038*	

### Atomic displacement parameters (Å^2^)

**Table:**

	*U*^11^	*U*^22^	*U*^33^	*U*^12^	*U*^13^	*U*^23^
S1	0.0408 (3)	0.0258 (3)	0.0297 (3)	0.0003 (3)	−0.0032 (2)	−0.0018 (3)
O1A	0.045 (6)	0.033 (4)	0.0448 (18)	−0.007 (5)	0.011 (5)	0.004 (2)
O1B	0.045 (6)	0.033 (4)	0.0448 (18)	−0.007 (5)	0.011 (5)	0.004 (2)
O2	0.0743 (17)	0.0657 (18)	0.0406 (12)	0.0136 (14)	−0.0202 (11)	−0.0143 (12)
O3	0.0636 (15)	0.0471 (14)	0.0538 (14)	0.0174 (12)	−0.0183 (12)	0.0023 (11)
N1	0.0326 (11)	0.0294 (12)	0.0260 (11)	−0.0036 (10)	0.0002 (8)	0.0008 (9)
N2	0.0416 (13)	0.0419 (15)	0.0318 (12)	−0.0038 (12)	−0.0050 (10)	0.0034 (11)
C1	0.0416 (16)	0.0271 (15)	0.0328 (14)	−0.0008 (13)	0.0061 (12)	0.0022 (12)
C2	0.0332 (13)	0.0263 (14)	0.0248 (12)	−0.0002 (10)	0.0061 (10)	0.0016 (10)
C3	0.0331 (13)	0.0318 (15)	0.0262 (12)	0.0040 (11)	0.0050 (10)	0.0013 (11)
C4A	0.047 (4)	0.035 (4)	0.036 (3)	0.009 (3)	0.002 (2)	0.004 (2)
C5A	0.044 (3)	0.052 (3)	0.034 (3)	0.013 (2)	−0.0027 (18)	0.005 (2)
C6A	0.040 (3)	0.054 (3)	0.034 (2)	0.005 (3)	−0.005 (2)	0.002 (2)
C7A	0.035 (4)	0.041 (4)	0.028 (3)	0.006 (3)	0.000 (2)	−0.007 (4)
C4B	0.047 (4)	0.035 (4)	0.036 (3)	0.009 (3)	0.002 (2)	0.004 (2)
C5B	0.044 (3)	0.052 (3)	0.034 (3)	0.013 (2)	−0.0027 (18)	0.005 (2)
C6B	0.040 (3)	0.054 (3)	0.034 (2)	0.005 (3)	−0.005 (2)	0.002 (2)
C7B	0.035 (4)	0.041 (4)	0.028 (3)	0.006 (3)	0.000 (2)	−0.007 (4)
C8	0.0310 (13)	0.0346 (16)	0.0274 (13)	0.0042 (11)	0.0025 (10)	0.0014 (11)
C9	0.0313 (13)	0.0247 (14)	0.0270 (13)	−0.0019 (10)	0.0035 (10)	−0.0015 (10)
C10A	0.021 (4)	0.025 (2)	0.0314 (16)	0.006 (3)	−0.008 (2)	0.0031 (15)
C11A	0.046 (4)	0.034 (3)	0.048 (2)	−0.008 (3)	0.000 (3)	−0.0066 (18)
C12A	0.057 (5)	0.040 (3)	0.050 (3)	−0.005 (3)	−0.010 (3)	−0.014 (2)
C13A	0.066 (5)	0.051 (4)	0.035 (2)	0.001 (4)	0.001 (3)	−0.016 (2)
C14A	0.065 (5)	0.047 (3)	0.041 (3)	−0.014 (4)	0.016 (3)	−0.011 (2)
C15A	0.036 (5)	0.033 (2)	0.036 (2)	−0.006 (3)	0.001 (3)	−0.0046 (18)
C10B	0.021 (4)	0.025 (2)	0.0314 (16)	0.006 (3)	−0.008 (2)	0.0031 (15)
C11B	0.046 (4)	0.034 (3)	0.048 (2)	−0.008 (3)	0.000 (3)	−0.0066 (18)
C12B	0.057 (5)	0.040 (3)	0.050 (3)	−0.005 (3)	−0.010 (3)	−0.014 (2)
C13B	0.066 (5)	0.051 (4)	0.035 (2)	0.001 (4)	0.001 (3)	−0.016 (2)
C14B	0.065 (5)	0.047 (3)	0.041 (3)	−0.014 (4)	0.016 (3)	−0.011 (2)
C15B	0.036 (5)	0.033 (2)	0.036 (2)	−0.006 (3)	0.001 (3)	−0.0046 (18)
C16	0.0319 (12)	0.0288 (15)	0.0279 (11)	−0.0029 (11)	0.0007 (9)	−0.0002 (11)
C17	0.0279 (12)	0.0257 (13)	0.0295 (12)	−0.0045 (10)	0.0009 (10)	0.0043 (10)
C18	0.0368 (13)	0.0284 (16)	0.0308 (13)	0.0034 (11)	0.0061 (10)	−0.0007 (10)
C19	0.0421 (14)	0.0324 (14)	0.0270 (11)	0.0005 (14)	0.0011 (10)	−0.0064 (13)
C20	0.0313 (13)	0.0319 (15)	0.0296 (12)	−0.0043 (11)	−0.0004 (10)	0.0039 (11)
C21	0.0332 (13)	0.0268 (13)	0.0330 (14)	−0.0008 (11)	0.0002 (10)	0.0016 (11)
C22	0.0375 (14)	0.0282 (14)	0.0292 (12)	−0.0039 (11)	−0.0001 (10)	−0.0031 (11)

### Geometric parameters (Å, º)

**Table:**

S1—C8	1.730 (3)	C7B—H7BA	0.9902
S1—C9	1.758 (3)	C7B—H7BB	0.9900
O1A—C1	1.213 (18)	C7B—C8	1.55 (4)
O1B—C1	1.27 (3)	C10A—C11A	1.3900
O2—N2	1.214 (4)	C10A—C15A	1.3900
O3—N2	1.221 (4)	C11A—H11A	0.9500
N1—C9	1.384 (3)	C11A—C12A	1.3900
N1—C16	1.282 (4)	C12A—H12A	0.9500
N2—C20	1.470 (3)	C12A—C13A	1.3900
C1—C2	1.487 (4)	C13A—H13A	0.9500
C1—C10A	1.499 (9)	C13A—C14A	1.3900
C1—C10B	1.493 (13)	C14A—H14A	0.9500
C2—C3	1.437 (4)	C14A—C15A	1.3900
C2—C9	1.378 (4)	C15A—H15A	0.9500
C3—C4A	1.51 (3)	C10B—C11B	1.3900
C3—C4B	1.50 (4)	C10B—C15B	1.3900
C3—C8	1.359 (4)	C11B—H11B	0.9500
C4A—H4AA	0.9898	C11B—C12B	1.3900
C4A—H4AB	0.9899	C12B—H12B	0.9500
C4A—C5A	1.537 (14)	C12B—C13B	1.3900
C5A—H5AA	0.9902	C13B—H13B	0.9500
C5A—H5AB	0.9899	C13B—C14B	1.3900
C5A—C6A	1.523 (9)	C14B—H14B	0.9500
C6A—H6AA	0.9900	C14B—C15B	1.3900
C6A—H6AB	0.9900	C15B—H15B	0.9500
C6A—C7A	1.550 (13)	C16—H16	0.9500
C7A—H7AA	0.9900	C16—C17	1.462 (3)
C7A—H7AB	0.9899	C17—C18	1.393 (4)
C7A—C8	1.48 (2)	C17—C22	1.396 (4)
C4B—H4BA	0.9900	C18—H18	0.9500
C4B—H4BB	0.9901	C18—C19	1.382 (4)
C4B—C5B	1.524 (17)	C19—H19	0.9500
C5B—H5BA	0.9900	C19—C20	1.389 (4)
C5B—H5BB	0.9899	C20—C21	1.377 (4)
C5B—C6B	1.511 (12)	C21—H21	0.9500
C6B—H6BA	0.9900	C21—C22	1.390 (4)
C6B—H6BB	0.9900	C22—H22	0.9500
C6B—C7B	1.553 (17)		
			
C8—S1—C9	91.40 (14)	C8—C7B—H7BB	113.4
C16—N1—C9	119.2 (2)	C3—C8—S1	112.3 (2)
O2—N2—O3	123.5 (3)	C3—C8—C7A	123.4 (5)
O2—N2—C20	118.4 (3)	C3—C8—C7B	130.5 (6)
O3—N2—C20	118.1 (2)	C7A—C8—S1	124.3 (5)
O1A—C1—C2	118.8 (9)	C7B—C8—S1	117.1 (6)
O1A—C1—C10A	117.4 (10)	N1—C9—S1	123.3 (2)
O1B—C1—C2	117.5 (13)	C2—C9—S1	110.8 (2)
O1B—C1—C10B	118.0 (15)	C2—C9—N1	125.8 (2)
C2—C1—C10A	121.2 (5)	C11A—C10A—C1	116.5 (7)
C2—C1—C10B	122.5 (7)	C11A—C10A—C15A	120.0
C3—C2—C1	122.2 (2)	C15A—C10A—C1	123.5 (7)
C9—C2—C1	125.0 (2)	C10A—C11A—H11A	120.0
C9—C2—C3	112.6 (2)	C12A—C11A—C10A	120.0
C2—C3—C4A	122.6 (5)	C12A—C11A—H11A	120.0
C2—C3—C4B	130.1 (8)	C11A—C12A—H12A	120.0
C8—C3—C2	112.9 (2)	C11A—C12A—C13A	120.0
C8—C3—C4A	124.5 (5)	C13A—C12A—H12A	120.0
C8—C3—C4B	117.0 (8)	C12A—C13A—H13A	120.0
C3—C4A—H4AA	110.8	C14A—C13A—C12A	120.0
C3—C4A—H4AB	108.5	C14A—C13A—H13A	120.0
C3—C4A—C5A	109.3 (14)	C13A—C14A—H14A	120.0
H4AA—C4A—H4AB	108.6	C15A—C14A—C13A	120.0
C5A—C4A—H4AA	110.8	C15A—C14A—H14A	120.0
C5A—C4A—H4AB	108.7	C10A—C15A—H15A	120.0
C4A—C5A—H5AA	110.6	C14A—C15A—C10A	120.0
C4A—C5A—H5AB	107.8	C14A—C15A—H15A	120.0
H5AA—C5A—H5AB	107.9	C11B—C10B—C1	119.7 (11)
C6A—C5A—C4A	111.7 (11)	C11B—C10B—C15B	120.0
C6A—C5A—H5AA	109.7	C15B—C10B—C1	119.6 (10)
C6A—C5A—H5AB	109.0	C10B—C11B—H11B	120.0
C5A—C6A—H6AA	109.7	C12B—C11B—C10B	120.0
C5A—C6A—H6AB	109.7	C12B—C11B—H11B	120.0
C5A—C6A—C7A	109.1 (9)	C11B—C12B—H12B	120.0
H6AA—C6A—H6AB	108.3	C13B—C12B—C11B	120.0
C7A—C6A—H6AA	111.8	C13B—C12B—H12B	120.0
C7A—C6A—H6AB	108.2	C12B—C13B—H13B	120.0
C6A—C7A—H7AA	107.9	C14B—C13B—C12B	120.0
C6A—C7A—H7AB	110.4	C14B—C13B—H13B	120.0
H7AA—C7A—H7AB	107.5	C13B—C14B—H14B	120.0
C8—C7A—C6A	110.0 (13)	C13B—C14B—C15B	120.0
C8—C7A—H7AA	109.1	C15B—C14B—H14B	120.0
C8—C7A—H7AB	111.9	C10B—C15B—H15B	120.0
C3—C4B—H4BA	106.4	C14B—C15B—C10B	120.0
C3—C4B—H4BB	111.5	C14B—C15B—H15B	120.0
C3—C4B—C5B	113 (2)	N1—C16—H16	119.0
H4BA—C4B—H4BB	107.7	N1—C16—C17	122.0 (3)
C5B—C4B—H4BA	106.4	C17—C16—H16	119.0
C5B—C4B—H4BB	111.0	C18—C17—C16	121.7 (2)
C4B—C5B—H5BA	107.1	C18—C17—C22	119.5 (2)
C4B—C5B—H5BB	110.3	C22—C17—C16	118.8 (2)
H5BA—C5B—H5BB	108.2	C17—C18—H18	119.7
C6B—C5B—C4B	112.0 (16)	C19—C18—C17	120.6 (3)
C6B—C5B—H5BA	110.4	C19—C18—H18	119.7
C6B—C5B—H5BB	108.8	C18—C19—H19	120.9
C5B—C6B—H6BA	108.9	C18—C19—C20	118.3 (2)
C5B—C6B—H6BB	109.6	C20—C19—H19	120.9
C5B—C6B—C7B	111.6 (15)	C19—C20—N2	118.6 (2)
H6BA—C6B—H6BB	107.7	C21—C20—N2	118.6 (3)
C7B—C6B—H6BA	106.1	C21—C20—C19	122.8 (2)
C7B—C6B—H6BB	112.8	C20—C21—H21	121.0
C6B—C7B—H7BA	107.1	C20—C21—C22	118.1 (3)
C6B—C7B—H7BB	113.8	C22—C21—H21	121.0
H7BA—C7B—H7BB	108.2	C17—C22—H22	119.6
C8—C7B—C6B	106.3 (17)	C21—C22—C17	120.7 (3)
C8—C7B—H7BA	107.7	C21—C22—H22	119.6
			
O1A—C1—C2—C3	40.0 (6)	C4B—C3—C8—S1	−178.4 (18)
O1A—C1—C2—C9	−133.7 (5)	C4B—C3—C8—C7A	4 (2)
O1A—C1—C10A—C11A	25.5 (8)	C4B—C3—C8—C7B	−3 (2)
O1A—C1—C10A—C15A	−155.8 (7)	C4B—C5B—C6B—C7B	63 (2)
O1A—C1—C10B—C11B	14.0 (11)	C5B—C6B—C7B—C8	−43 (2)
O1A—C1—C10B—C15B	−157.1 (8)	C6B—C7B—C8—S1	−169.6 (11)
O1B—C1—C2—C3	60.5 (10)	C6B—C7B—C8—C3	16 (3)
O1B—C1—C2—C9	−113.2 (10)	C6B—C7B—C8—C7A	−25 (10)
O1B—C1—C10A—C11A	5.2 (11)	C8—S1—C9—N1	174.9 (2)
O1B—C1—C10A—C15A	−176.1 (10)	C8—S1—C9—C2	−0.8 (2)
O1B—C1—C10B—C11B	−6.6 (13)	C8—C3—C4A—C5A	−16 (2)
O1B—C1—C10B—C15B	−177.7 (11)	C8—C3—C4B—C5B	19 (3)
O2—N2—C20—C19	1.1 (4)	C9—S1—C8—C3	−1.1 (2)
O2—N2—C20—C21	−179.3 (3)	C9—S1—C8—C7A	176.7 (11)
O3—N2—C20—C19	179.9 (3)	C9—S1—C8—C7B	−176.8 (14)
O3—N2—C20—C21	−0.5 (4)	C9—N1—C16—C17	−179.2 (2)
N1—C16—C17—C18	−12.3 (4)	C9—C2—C3—C4A	175.7 (13)
N1—C16—C17—C22	166.3 (3)	C9—C2—C3—C4B	178 (2)
N2—C20—C21—C22	−179.6 (2)	C9—C2—C3—C8	−3.2 (3)
C1—C2—C3—C4A	1.3 (14)	C10A—C1—C2—C3	−121.2 (4)
C1—C2—C3—C4B	4 (2)	C10A—C1—C2—C9	65.1 (5)
C1—C2—C3—C8	−177.6 (3)	C10A—C1—C10B—C11B	102 (5)
C1—C2—C9—S1	176.5 (2)	C10A—C1—C10B—C15B	−69 (5)
C1—C2—C9—N1	1.0 (4)	C10A—C11A—C12A—C13A	0.0
C1—C10A—C11A—C12A	178.7 (6)	C11A—C10A—C15A—C14A	0.0
C1—C10A—C15A—C14A	−178.7 (7)	C11A—C12A—C13A—C14A	0.0
C1—C10B—C11B—C12B	−171.1 (11)	C12A—C13A—C14A—C15A	0.0
C1—C10B—C15B—C14B	171.1 (11)	C13A—C14A—C15A—C10A	0.0
C2—C1—C10A—C11A	−173.1 (3)	C15A—C10A—C11A—C12A	0.0
C2—C1—C10A—C15A	5.6 (7)	C10B—C1—C2—C3	−135.7 (7)
C2—C1—C10B—C11B	−170.3 (5)	C10B—C1—C2—C9	50.6 (7)
C2—C1—C10B—C15B	18.6 (11)	C10B—C1—C10A—C11A	−73 (5)
C2—C3—C4A—C5A	164.9 (8)	C10B—C1—C10A—C15A	105 (5)
C2—C3—C4B—C5B	−162.1 (11)	C10B—C11B—C12B—C13B	0.0
C2—C3—C8—S1	2.6 (3)	C11B—C10B—C15B—C14B	0.0
C2—C3—C8—C7A	−175.2 (11)	C11B—C12B—C13B—C14B	0.0
C2—C3—C8—C7B	177.6 (17)	C12B—C13B—C14B—C15B	0.0
C3—C2—C9—S1	2.3 (3)	C13B—C14B—C15B—C10B	0.0
C3—C2—C9—N1	−173.3 (2)	C15B—C10B—C11B—C12B	0.0
C3—C4A—C5A—C6A	45.5 (19)	C16—N1—C9—S1	−14.8 (4)
C3—C4B—C5B—C6B	−49 (3)	C16—N1—C9—C2	160.3 (3)
C4A—C3—C4B—C5B	−148 (21)	C16—C17—C18—C19	178.7 (2)
C4A—C3—C8—S1	−176.3 (13)	C16—C17—C22—C21	−178.1 (2)
C4A—C3—C8—C7A	5.9 (17)	C17—C18—C19—C20	−0.8 (4)
C4A—C3—C8—C7B	−1 (2)	C18—C17—C22—C21	0.5 (4)
C4A—C5A—C6A—C7A	−65.1 (16)	C18—C19—C20—N2	−179.7 (2)
C5A—C6A—C7A—C8	51.0 (15)	C18—C19—C20—C21	0.8 (4)
C6A—C7A—C8—S1	159.4 (6)	C19—C20—C21—C22	−0.1 (4)
C6A—C7A—C8—C3	−23.0 (18)	C20—C21—C22—C17	−0.6 (4)
C6A—C7A—C8—C7B	120 (13)	C22—C17—C18—C19	0.2 (4)
C4B—C3—C4A—C5A	−2 (18)		

### Hydrogen-bond geometry (Å, º)

**Table:**

*D*—H···*A*	*D*—H	H···*A*	*D*···*A*	*D*—H···*A*
C4*A*—H4*AA*···O1*B*^i^	0.99	2.38	3.15 (4)	135
C4*B*—H4*BA*···O1*A*^i^	0.99	2.50	3.45 (4)	162
C4*B*—H4*BA*···O1*B*^i^	0.99	2.26	3.18 (4)	154
C7*B*—H7*BB*···O2^ii^	0.99	2.46	3.44 (3)	169
C13*B*—H13*B*···O3^iii^	0.95	2.55	3.371 (13)	145
C21—H21···O1*A*^iv^	0.95	2.40	3.127 (18)	133
C21—H21···O1*B*^iv^	0.95	2.44	3.13 (3)	129

Symmetry codes: (i) *x*+1, *y*, *z*; (ii) *x*+1, *y*, *z*+1; (iii) −*x*−1, *y*−1/2, −*z*; (iv) −*x*−1, *y*+1/2, −*z*+1.
